# *Astragalus membranaceus*-Polysaccharides Ameliorates Obesity, Hepatic Steatosis, Neuroinflammation and Cognition Impairment without Affecting Amyloid Deposition in Metabolically Stressed APPswe/PS1dE9 Mice

**DOI:** 10.3390/ijms18122746

**Published:** 2017-12-18

**Authors:** Yung-Cheng Huang, Huey-Jen Tsay, Mei-Kuang Lu, Chien-Hung Lin, Chih-Wen Yeh, Hui-Kang Liu, Young-Ji Shiao

**Affiliations:** 1 Department of Physical Medicine and Rehabilitation, Cheng Hsin General Hospital, Taipei 11220, Taiwan; jeremy0681@gmail.com; 2 Program in Molecular Medicine, School of Life Sciences, National Yang-Ming University, Taipei 11221, Taiwan; 3 Institute of Neuroscience, Brain Research Center, School of Life Science, National Yang-Ming University, Taipei 11221, Taiwan; hjtsay@ym.edu.tw (H.-J.T.); whitebrake@hotmail.com (C.-H.L.); flow5168@hotmail.com (C.-W.Y.); 4 National Research Institute of Chinese Medicine, Ministry of Health and Welfare, Taipei 11221, Taiwan; mklu@nricm.edu.tw; 5 Program in Clinical Drug Development of Chinese Herbal Medicine, Taipei Medical University, Taipei 11031, Taiwan; 6 Institute of Biopharmaceutical Science, National Yang-Ming University, Taipei 11221, Taiwan

**Keywords:** cognitive dysfunction, Astragalus-polysaccharides, glia, metabolic stresses, amyloid plaque, Alzheimer’s disease

## Abstract

*Astragalus membranaceus* is commonly used in traditional Chinese medicine for strengthening the host defense system. *Astragalus membranaceus*-polysaccharides is an effective component with various important bioactivities, such as immunomodulation, antioxidant, anti-diabetes, anti-inflammation and neuroprotection. In the present study, we determine the effects of *Astragalus membranaceus*-polysaccharides on metabolically stressed transgenic mice in order to develop this macromolecules for treatment of sporadic Alzheimer’s disease, a neurodegenerative disease with metabolic risk factors. Transgenic mice, at 10 weeks old prior to the appearance of senile plaques, were treated in combination of administrating high-fat diet and injecting low-dose streptozotocin to create the metabolically stressed mice model. *Astragalus membranaceus*-polysaccharides was administrated starting at 14 weeks for 7 weeks. We found that *Astragalus membranaceus*-polysaccharides reduced metabolic stress-induced increase of body weight, insulin and insulin and leptin level, insulin resistance, and hepatic triglyceride. *Astragalus membranaceus*-polysaccharides also ameliorated metabolic stress-exacerbated oral glucose intolerance, although the fasting blood glucose was only temporally reduced. In brain, metabolic stress-elicited astrogliosis and microglia activation in the vicinity of plaques was also diminished by *Astragalus membranaceus*-polysaccharides administration. The plaque deposition, however, was not significantly affected by *Astragalus membranaceus*-polysaccharides administration. These findings suggest that *Astragalus membranaceus*-polysaccharides may be used to ameliorate metabolic stress-induced diabesity and the subsequent neuroinflammation, which improved the behavior performance in metabolically stressed transgenic mice.

## 1. Introduction

Sporadic Alzheimer’s disease (AD) is a metabolic disease relative to impairments in brain insulin signaling and energy metabolism, which lead to increased oxidative stress, inflammation, and aggravation of insulin resistance [1]. Epidemiological research suggests that type II diabetes mellitus (T2DM) increases the risk of AD. Multiple possible links between T2DM and sporadic AD including insulin resistance, inflammation, and oxidative stress have been proposed. Therefore, metabolic stress may directly contribute to loss of neuron and synaptic connection, tau hyperphosphorylation, and amyloid-β (Aβ) accumulation in AD [1]. In addition, chronic inflammation mediated by over activation of microglia and/or astrocytes in brain is involved in AD pathogenesis [2].

In our previous studies, we found that obesity, hyperglycemia, hepatic steatosis, Aβ plaque burdens and cerebrovascular inflammation in APPswe/PS1ΔE9 (APP/PS1) transgenic mice is accelerated by the combination of high-fat diet (HFD) and a low-dose injection of streptozotocin (STZ) (i.e., HFSTZ AD mice) [3,4,5]. In those studies, we found an interplay between genetic background of AD and HFSTZ-induced metabolic stress. Moreover, HFSTZ aggravated vascular inflammation, astrocyte activation, and impairment of cerebral glucose metabolism and daily living activity in APP/PS1 mice.

The root of *Astragalus membranaceus* is an herb commonly used in traditional Chinese medicine for strengthening the host defense system [6]. The bioactive constituents in the dried root of *A. membranaceus* are complicated, containing polysaccharides, flavonoids, astragalosides, etc. [7]. *A. membranaceus* polysaccharides (APS) was identified as one of the major active ingredients responsible for the bioactivities, including antioxidant, immunomodulation, anti-inflammation, anti-diabetes, anti-atherosclerosis, hematopoiesis, hepatoprotection and neuroprotection [8,9,10,11]. Moreover, APS has also been found to decrease the body weight and blood glucose, and improve the insulin sensitivity in the muscle of HFD plus STZ-induced T2DM rats [12] and mice [13], and improvement of early diabetic nephropathy in T2DM rats [14]. However, the effect of APS on metabolic stressed AD model has not yet been investigated.

In this study, we aim to examine the effect of APS on metabolic stress-aggravated pathological progression of AD. We hypothesized that APS may ameliorate diabesity-associated metabolic changes on cerebral and hepatic inflammation that in turn reduce neuroinflammation-related pathologies. We examined blood glucose, insulin, homeostasis model assessment of insulin resistance (HOMA-IR), Leptin, body weight, epididymal fat, hepatic steatosis, Aβ burden, glial activation, and nesting behavior in HFSTZ-APP/PS1 mice.

## 2. Results

### 2.1. Characterization of Astragalus membranaceus Polysaccharides (APS)

**Figure 1:**
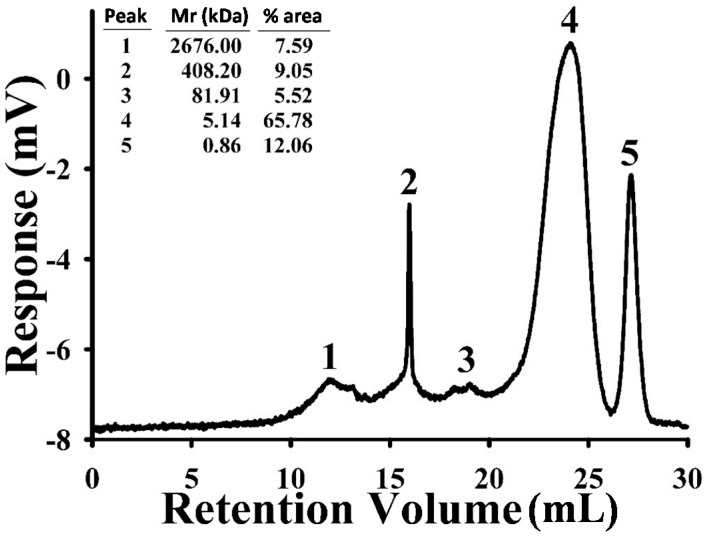
The composition of *Astragalus membranaceus* Polysaccharides (APS). Carbohydrate solution was injected onto the Size-Exclusion Chromatography (SEC) column and was eluted by deionized water. SEC signal detection (response, mV) was performed using a ViscoTek model TDA-3-1 relative viscometer (Viscotek, Houston, TX, USA). Sodex P-82 series (Showa Denko America, New York, NY, USA) containing polymaltotriose with molecular weights of 78.8 × 10^4^, 40.4 × 10^4^, 21.2 × 10^4^, 4.73 × 10^4^, and 1.18 × 10^4^ Daltons (Da) was used as authentic standard. The molecular weight (Mr (kDa)) and area percentage (% area) of each peak was calculated and displayed at upper left corner.

For studying the effects of APS on metabolically stressed AD, APS was isolated. Lyophilized dried roots of *Astragalus membranaceus* were extracted with water and precipitated with 95% ethanol. After lyophilization, a crude brownish polysaccharide denoted as APS was obtained. Yield of APS was determined as 3.47 ± 0.08%. The molecular mass distribution of APS was determined by chromatography (Figure 1). The result showed that there were five populations of APS with molecular weight range from 2676.00 (peak 1) to 0.86 kDa (peak 5). The major polysaccharide population was low-molecular-weight polysaccharides (5.14 kDa, peak 4) in the percent area 65.78. The major monosaccharide composition of APS is fucose:myo-inositol:fructose:sorbitol:glucose in the ratio of 1:1.4:2.1:13.7:91.5.

### 2.2. APS Ameliorates HFSTZ-Induced Insulin Resistance and Hyperleptinemia

**Figure 2:**
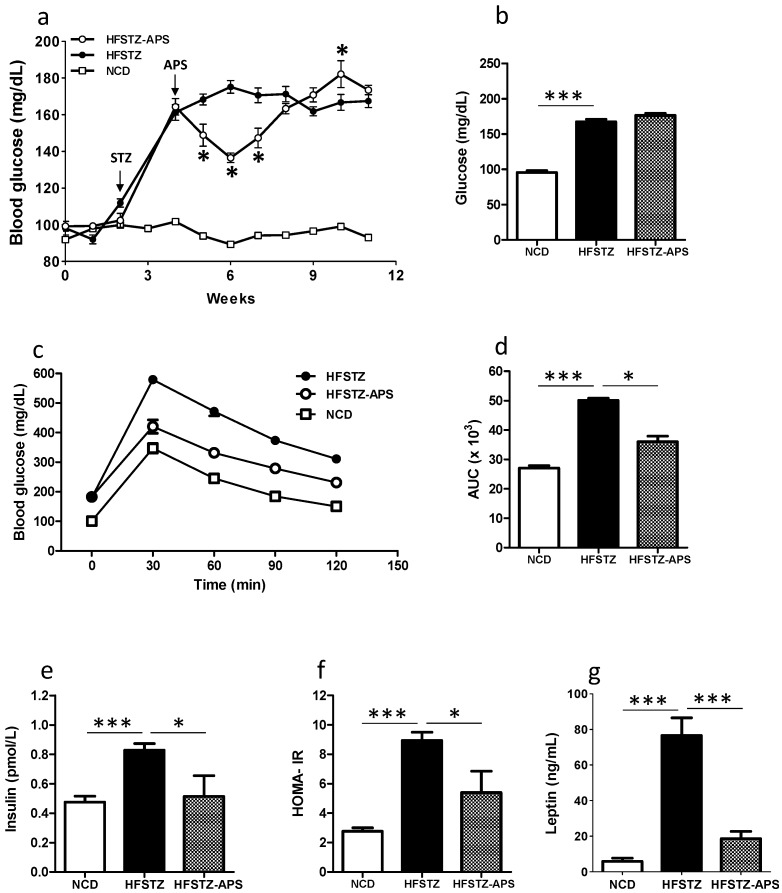
APS ameliorated HFSTZ-impacted glycemic control. Three groups of APP/PS1 mice: normal chow diet (NCD) (*n* = 6), high fat diet plus streptozotocine (HFSTZ) (*n* = 7) or HFSTZ plus APS (HFSTZ-APS) (*n* = 6) are examined. (**a**) Fasting glucose levels detected every week; (**b**) Fasting glucose levels detected at 11 weeks after dietary manipulation; (**c**) Oral glucose tolerance testing was performed 10 weeks after dietary manipulation; (**d**) Area under curve (AUC) values of panel c; (**e**) Fasting insulin; (**f**) homeostasis model assessment of insulin resistance (HOMA-IR), and (**g**) leptin were determined at 11 weeks after dietary manipulation. Bars represent the mean ± SEM. * *p* < 0.01 and *** *p* < 0.001, significant difference from HFSTZ group.

APS are the key components of *Astragalus membranaceus*, which capable to improve insulin resistance and reduced blood glucose is widely used in Traditional Chinese Medicine [15]. Whether APS ameliorates hyperglycemia of HFSTZ AD mice was evaluated. HFSTZ induced a significant increase level of fasting blood glucose in APP/PS1 transgenic mice after 4 weeks administration of HFD (i.e., 2 weeks after STZ injection) (Figure 2a). There is only transient reduction on HFSTZ-induced hyperglycemia during the first three weeks after APS administration (Figure 2a). At the 11th week, however, the fasting blood glucose was not significant different between HFSTZ and normal chow diet (NCD) groups (Figure 2a,b). Oral glucose tolerance test (OGTT) was performed at 10 weeks post HFD administration. The result showed that APS reduced the HFSTZ-induced glucose intolerance (Figure 2c). The significant difference was confirmed by comparing the lower area under the curve (AUC) of glucose tolerance assay (Figure 2d). For serum insulin concentration, we observed that HFSTZ-induced hyperinsulinemia (increased insulin level) and insulin resistance (elevated HOMA-IR) were mitigated by APS (Figure 2e,f). For serum leptin concentration, we observed that HFSTZ-induced hyperleptinemia (increased leptin level) was also alleviated by APS (Figure 2g).

### 2.3. APS Ameliorates HFSTZ-Induced Obesity and Hepatic Steatosis, but Not Adipocyte Hypertrophy

**Figure 3:**
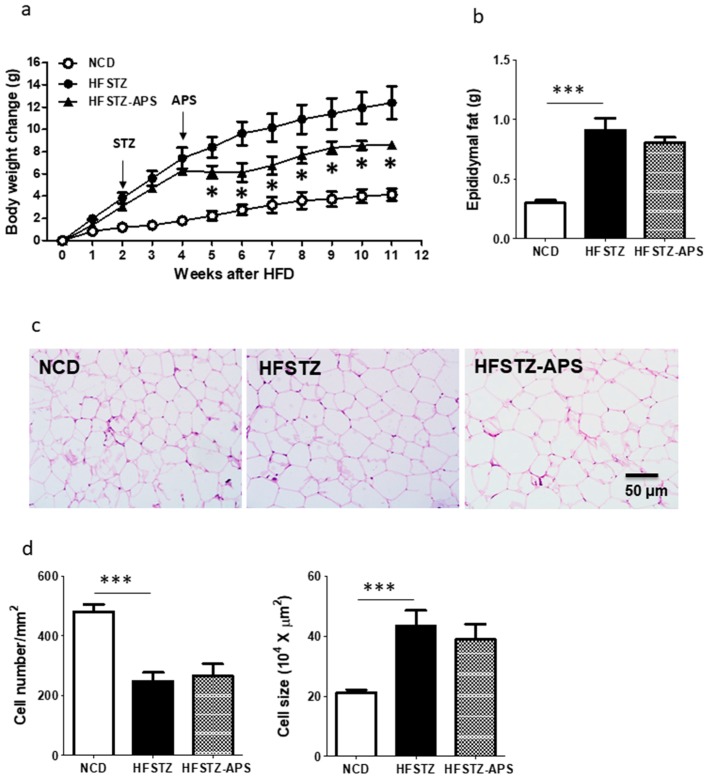
APS recovered the body weight increase mediated by the treatment of HFSTZ. Three groups of APP/PS1 mice: NCD (*n* = 6), HFSTZ (*n* = 7) or HFSTZ-APS (*n* = 6) are examined. (**a**) Body weights were recorded every week; (**b**) The mass of epididymal fat tissue was determined; (**c**) Representative histological microphotographs of hematoxylinand eosin (HE)-stained epididymalfat sections were shown (scale bar, 50 μm); (**d**) Adipocyte number and size were measured by ImageJ. Bars represent the mean ± SEM of atleast three independent experiments. Bars represent the mean ± SEM. * *p* < 0.01 and *** *p* < 0.001, significant difference from HFSTZ group.

As shown in Figure 3a, HFSTZ-induced obesity was significantly reduced by the administration of APS. At the end of experiment, the epididymal fat weight was significantly increased and adipocyte size was significantly enlarged in HFSTZ mice as compared with NCD mice. However, APS was ineffective on reducing this adipocyte hypertrophy including epididymal fat mass, and adipocyte number and size (Figure 3b–d).

**Figure 4:**
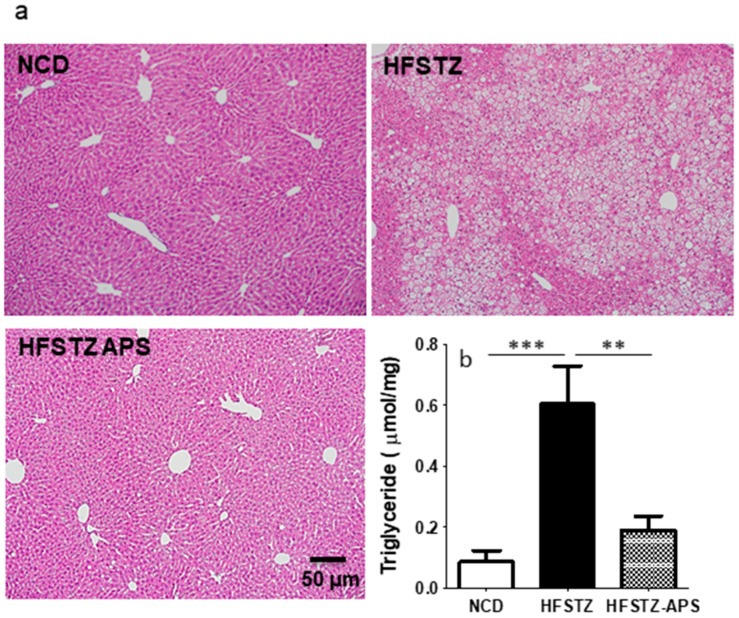
APS ameliorated HFSTZ-induced hepatic steatosis. Three groups of APP/PS1 mice: NCD (*n* = 6), HFSTZ (*n* = 5) or HFSTZ-APS (*n* = 7) are examined. (**a**) Representative images of HE-stained liver sections (scale bar, 50 μm) were shown. (**b**) Hepatic triacyl glycerol (TG) contents were quantified. Bars represent the mean ± SEM. ** *p* < 0.01 and *** *p* < 0.001, significant difference from HFSTZ group.

In hematoxylinand eosin (HE)-stained liver section, severe vacuolation of hepatocytes was observed in HFSTZ mice (Figure 4a). We found that APS significantly reduce this vacuolation of hepatocytes. Consistently, HFSTZ-increased liver triacyl glycerol (TG) content was mitigated by APS (Figure 4b).

### 2.4. APS Did not Significantly Reduce HFSTZ-Aggravated Cerebral Aβ Deposition 

**Figure 5:**
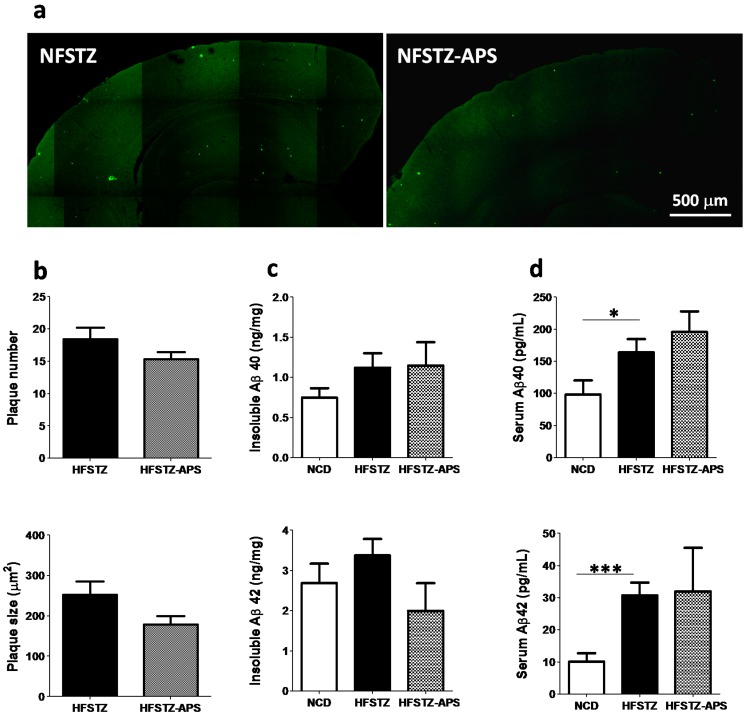
APS did not ameliorate HFSTZ-aggravated cerebral Aβ deposition and serum Aβ. APP/PS1 mice were treated with HFSTZ (*n* = 5) or HFSTZ-APS (*n* = 6); (**a**) Representative images of 1-bromo-2,5-bis-(3-hydroxycarbonyl-4-hydroxy) styrylbenzene (BSB)-positive plaques in the cerebral cortex of HFSTZ and HFSTZ-APS mice (scale bar, 500 μm); (**b**) Plaque size and plaque number in a single hemisphere were calculated using Metamorph analysis software. APP/PS1 mice were treated with NCD (*n* = 14), HFSTZ (*n* = 15) or HFSTZ-APS (*n* = 7); (**c**) Levels of soluble and insoluble form Aβ40 and Aβ42 in cerebral cortex were measured by enzyme-linked immunosorbent assay (ELISA); (**d**) The Serum Aβ40 and Aβ42 levels were measured using ELISA. Bars represent the mean ± SEM of four independent experiments. Data are shown as mean ± SEM. * *p* < 0.05, and *** *p* < 0.001, significant difference from HFSTZ group.

BSB ((*trans*, *trans*),-1-bromo-2,5-bis-(3-hydroxycarbonyl-4-hydroxy) styrylbenzene) was used to stain senile plaque and the effect of APS on plaque accumulation was analyzed. The result did not show any significant effect of APS on the burden of cerebral Aβ plaques in HFSTZ mice (Figure 5a,b). Using an enzyme-linked immunosorbent assay (ELISA) assay to determine the level of Aβ in both cerebrum and serum. The result, again, did not show any significant effect of APS on the level of Aβ (Figure 5c,d).

### 2.5. APS Diminished HFSTZ-Activated Plaque-Associated Astrocytes and Microglia

**Figure 6:**
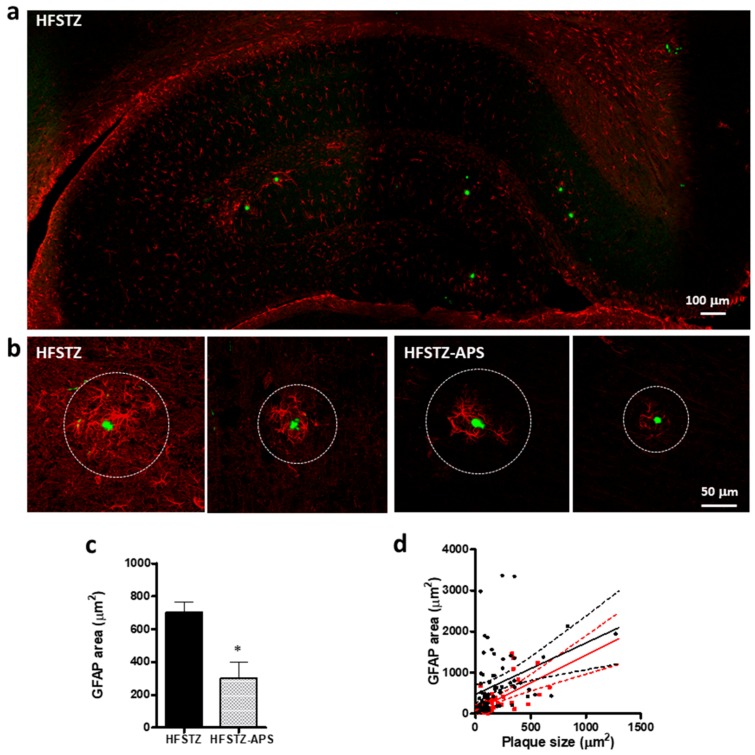
APS ameliorated HFSTZ-augmented activation of astrocytes in the vicinity of plaques. APP/PS1 mice were treated with HFSTZ (*n* = 4) or HFSTZ-APS (*n* = 4). (**a**) Representative confocal images of plaque (green) and the plaque-associated astrocytes immunostained with anti-GFAP (red) antibody were shown. Scale bar, 100 μm; (**b**) Representative confocal images of two selected plaques (green) surrounded by GFAP-positive astrocytes (red) in HFSTZ and HFSTZ-APS mice. Scale bar, 50 μm; (**c**) GFAP-positive astrocyte area in the dotted circle indicated in panel b (8 time diameter of the surrounded plaque) in the vicinity of plaques in HFSTZ and HFSTZ-APS mice. Data are shown as mean ± SEM. * *p* < 0.05, significant difference from HFSTZ group; (**d**) The correlation of plaque size and GFAP positive area was analyzed by a scatter plot from 72 and 21 plaques in HFSTZ (black) and HFSTZ-APS (red) mice, respectively. Solid lines are linear regression lines and dashed lines are 95% confidence intervals, *R*^2^ = 0.13 for HFSTZ group and *R*^2^ = 0.17 for HFSTZ-APS group.

The fluorescence intensity of glial fibrillary acidic protein (GFAP) was determined to assess the reactivity of plaque-associated astrocytes in an area with 8 times the diameter of the plaques which the astrocyte surrounded. The result shows that APS significantly diminished the reactivity of plaque-associated astrocytes (Figure 6a–c). A scatter plot of plaque size and GFAP area from 72 and 64 plaques in HFSTZ and HFSTZ-APS mice, respectively, was plotted to reveal the change of the activated astrocytes in the vicinity of plaque after APS treatment. The result showed that the APS treatment diminished HFSTZ-induced activation of plaque-associated astrocytes (Figure 6d). Linear regression for HFSTZ group is *y* = 1.25*x* + 470.6, *R*^2^ = 0.13, for HFSTZ-XZD group is *y* = 1.35*x* + 78.39, *R*^2^ = 0.37 (*p* = 0.88 for difference in slops; *p* < 0.001 for difference in intercept), suggesting that the effect is plaque size-independent.

**Figure 7:**
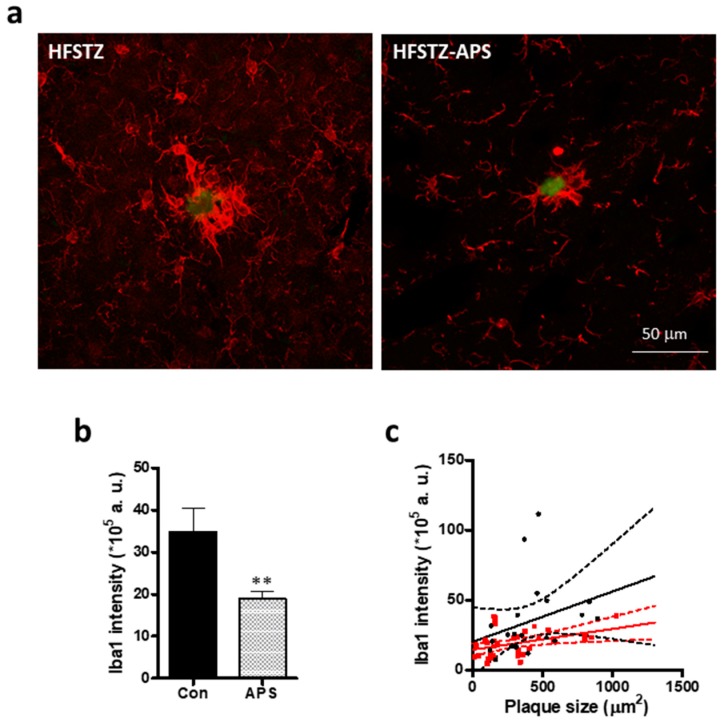
APS ameliorated HFSTZ-augmented activation of plaque-associated microglia. APP/PS1 mice were treated with HFSTZ (*n* = 4) or HFSTZ-APS (*n* = 4). (**a**) Representative images of the plaque-associated microglia immunostained with anti-Iba-1 antibody. Scale bar, 50 μm; (**b**) Immunoreactivity of Iba-1 in plaque-associated microglia in HFSTZ and HFSTZ-APS mice. Data are shown as mean ± SEM. ** *p* < 0.001, significant difference from HFSTZ group; (**c**) The correlation of plaque size and Iba1 intensity was analyzed by a scatter plot of plaque-associated microglia and plaque size from the selected plaques with vehicle (black) and APS (red) treatments. Solid lines is linear regression lines and dashed lines are 95% confidence intervals, *R*^2^ = 0.33 for HFSTZ group and *R*^2^ = 0.61 for HFSTZ-APS group.

Ionized calcium-binding adaptor molecule-1 (Iba-1) was used as microglia markers. We observed that microglia directly contacted the plaques. APS diminished the immunoreactivity of the Iba-1 that was associated with plaque. (Figure 7a,b). Plaque size and Iba-1 fluorescence intensity was analyzed by a scatter plot of 57 plaques to reveal the change of the activated plaque-associated microglia after APS treatment. The result showed that the APS treatment diminished HFSTZ-activated plaque-associated microglia (Figure 7c). Linear regression for HFSTZ group is *y* = 0.04*x* + 20.16, *R*^2^ = 0.33, for HFSTZ-APS group is *y* = 0.01*x* + 14.62, *R*^2^ = 0.17 (*p* = 0.30 for difference in slops; *p* < 0.01 for difference in intercept), suggesting that the effect is plaque size-independent.

### 2.6. APS Ameliorated HFSTZ-Prolonged Time for Nest Construction in AD Mice

**Figure 8:**
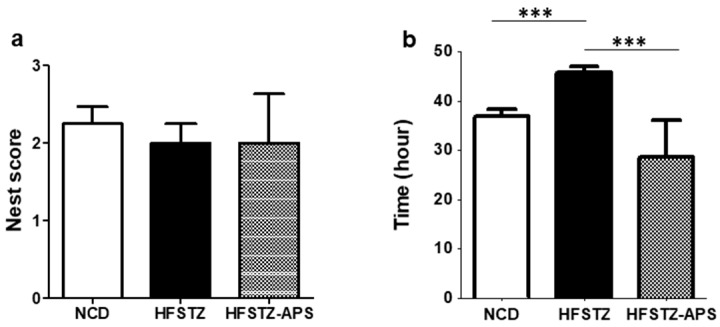
APS improved HFSTZ-prolonged nesting construction time. Nesting behavior for NCD (*n* = 7), HFSTZ (*n* = 6), and HFSTZ-APS (*n* = 3) mice groups were examined. (**a**) Nesting score (**b**) Nestcomplete-construction time. Data are shown as mean ± SEM. *** *p* < 0.001, significant difference from HFSTZ group.

A broad regions of brain has involved in nesting behavior of mice. The nesting task is therefore been used to evaluate cognition in AD animal models [3,5,16]. The result showed that the nesting score cannot tell the difference on the ability of nest construction among various mice groups. However, APS treatment recovered the prolonged nest constructing time for the HFSTZ mice (Figure 8).

## 3. Discussion

Cerebrovascular inflammation and glucose hypometabolism were advised to associate with the cognitive impairment in diabesity [17]. Diet-induced peripheral metabolic changes have been suggested to be linked with amyloidosis [18]. Previously, we created an HFSTZ APP/PS1 animal model, which displayed diabesity and hepatic steatosis; elevated peripheral Aβ level; enhanced Aβ level, plaque burden, astrocyte activation, vascular inflammation, glucose hypometabolism in the cerebrum, and caused cognitive impairment in APP/PS1 mice [3,4]. In the present study, the effects of APS on the pathological changes stated above were studied. We found that HFSTZ-mediated diabesity, leptinemia, and hepatic steatosis were significantly alleviated by APS. However, APS did not affect adipocyte hypertrophy and only transient reduction of HFSTZ-induced hyperglycemia. In the brain, the Aβ-relative pathological changes were inspected. The result showed that APS reduced astrogliosis and microglia activation and ameliorated the nesting activity. Moreover, APS also alleviated HFSTZ-aggravated hepatic steatosis. However, there was no effect APS on serum level of Aβ.

Following administration of APS (500 mg/kg/day) for 7 weeks, there was no difference in fasting blood glucose level and mass of epididymal fat. However, it is clear that APS markedly reduced HFSTZ-altered body weight, fasting serum insulin level, MOMA-IR, serum leptin level, hepatic TG and hepatic steatosis. These results suggest that the effects of APS may be specific to vessel leading to anti-inflammation property, since diabetes and obesity have been indicated to induce hepatic oxidation and inflammation [19]. The increased leptin level and increased body weight might implicate that leptin resistance was induced by HFZTZ treatment (4). Recent study indicated that APS altered glucose metabolism and decreased insulin resistance in rat with STZ-induced diabetes [20]. In that work, the potential mechanism of APS on glucose and lipid metabolism, anti-oxidative, insulin resistance and memory deficit in diabetes was studied. In those works, APS (200–800 mg/kg/day) was administrated for 8 weeks, and the fasting plasma glucose was significantly reduced after 8 weeks administration of APS as compared with the model group. In our present study, the fasting blood glucose was significantly reduced after 1–3 weeks, but not 4–7 weeks, administration of APS as compared with the model group. This different may be attributed to that the genetic background of APP/PS1 mice affects peripheral metabolism in the context of diabesity [4]. The similar result was also found in another previous work [5].

Liver has been suggested to be the major organ for plasma Aβ clearance [21]. Therefore, the elevated peripheral Aβ in HFSTZ mice might be attributed to hepatic steatosis. In our previous study [4], liver degradation play an important role on regulating the level of peripheral Aβ which may in turn affect Aβ drain out from CNS [21]. Our previous study suggested that obesity and hepatic steatosis were associated with elevated both serum Aβ42 and Aβ40, and the amount of serum Aβ42 was reduced by the administration of Xuefu Zhuyu decoction, a traditional Chinese classical herbal formula used for promoting blood circulation and removing blood stasis [4,5]. In the present study, however, we found that HFSTZ-aggravated level of both serum Aβ42 and Aβ40 were not ameliorated by APS. This result suggests that Aβ42-specific degradation in peripheral was not activated by APS, which does not promote blood circulation.

Growing evidence suggested that metabolic stressimpedes the functioning of the cerebrovascular neuroinflammation and Aβ deposition in AD mouse models. The diabesity-mediated cognitive impairments were suggested to be associated with altering the integrity and transport functions of blood-brain barrier (BBB), inducing oxidative stress and inflammation in the microcapillaries of central nervous system (CNS) [17] which subsequently results in Aβ overproduction and/or insufficient clearance. In turn, the accumulated Aβ may further induces neuroinflammation and neurotoxicity [2]. A defective vascular system was reported to reduce the drainage of parenchyma Aβ [21]. Therefore, a vicious cycle between vascular impairment and Aβ-related neurotoxicity is formed during AD pathogenesis progression. In the present study, HFSTZ-aggravated cerebral Aβ deposition was not reduced by APS. The nesting activity of mice, however, is improved, suggesting that cerebral Aβ deposition may not the major factor to influence nesting behavior.

Another histological feature of AD is the present and accumulation of reactive astrocytes and microglia around plaque, which is called gliosis or neuroinflammation [22]. Previously, APS has been found to be a promising therapeutic strategy for reducing inflammatory response [23] and possess anti-inflammatory activity [24,25,26,27]. Gliosis was observed to evaluate the effect of APS on ameliorating HFSTZ-induced neuroinflammation in our present study. 

The roles of astrocytes such as glucose transportation; neuronal homeostasis and metabolism modulation; blood-brain barrier maintain; and microenvironment sensing are important for brain physiological function regulation. Thus, the over-activated astrocytes are functionally impaired [28]. Aβ plaque-associated reactive astrocytes displayed upregulating of glial fibrillary acidic protein (GFAP) expression and pro-inflammatory mediator production, suggesting the detrimental role of reactive astrocytes in AD [29]. Therefore, as shown in a previous study, Aβ burden, microglial activation, and cognitive impairment may be decreased by suppressing astrocyte activation in a mouse model of AD [30]. Our present study found that APS significantly ameliorate HFSTZ-aggravated astrocyte reactivity in APP/PS1 mice, which is not correlated with plaque size, suggesting a direct effect of APS on astrocyte reactivity. We detected the reactivity of plaque-associated astrocytes by GFAP-positive area in 8 times diameter of the surrounded plaque, since the activated astrocytes did not contact with the plaque but surround plaque in a distance. For microglia, however, is directly contact with plaque. Therefore, the plaque-associated Iba-1-immunoreactivity was determined. In addition, this quantification cannot be replaced by Western Blotting since it is difficult to isolate plaque from the brains of mice.

Microglia mediate neurotoxicity in AD [2]. Aβ-activated microglia not only induced pro-inflammatory activity [31] but also contribute to plaque growth [32], suggesting that plaque-associated microglia is one of the mediators in AD pathology. Our present study found that APS significantly ameliorate the HFSTZ-aggravated plaque-associate microglia activation in APP/PS1 mice, which is independent with plaque size, suggesting the indirect effect of APS on microglia activity, and astrocyte activation may be the mediator. The effects of APS on HFSTZ-aggravated glial activation in APP/PS1 mice may include reducing astrocyte activation and mitigating microglia activation, which may affect nesting activity directly.

For mice, nesting behavior may be regarded as goal-directed. Several brain regions, including the hippocampus, are involved for nest construction [33]. Thus, nest construction task can be employed to evaluate the daily living activity of mice [34]. Previously, we have indicated that APP/PS1 mice were impaired in nesting construction by increasing the nesting construction time, but not the nest score [3]. These results may be responsible of that 16 h for nest scoring cannot differentiate the behavior impairment between NCD-APP/PS1 mice and HFSTZ-APP/PS1 mice. Therefore, the nest-completing time was employed. Our present results suggest that APS significantly decreased the nesting construction time, but not the nest score, in HFSTZ-APP/PS1 mice. This result suggested that APS recovered the nesting construction time prolonged by HFSTZ treatment.

## 4. Materials and Methods

### 4.1. Isolation of Polysaccharides

Lyophilized dried roots of *Astragalus membranaceus* were extracted with 80 °C water (1:20 (*w*/*w*)) for 6 h twice. After cooling, four volumes of 95% ethanol are added into the extract to precipitate polysaccharides overnight at 4 °C. The precipitated were collected by centrifugation. After lyophilization, a crude brownish polysaccharide denoted as APS was obtained.

### 4.2. Size-Exclusion Chromatography (SEC) 

Carbohydrate solution (1 mg/mL) was filtered through a 0.22 μm filter (Millipore, MA, USA) and then injected onto the SEC column. The flow rate was 0.5 mL/min, with deionized water being used as the eluent. A calibration curve was constructed using an authentic standard, Sodex P-82 series (Showa Denko America, New York, NY, USA) containing polymaltotriose with molecular weights of 78.8 × 10^4^, 40.4 × 10^4^, 21.2 × 10^4^, 4.73 × 10^4^, and 1.18 × 10^4^ Daltons (Da). The TriSec software program was used for the acquisition and analysis of Viscotek data. SEC signal detection was performed using a ViscoTek model TDA-3-1 relative viscometer (Viscotek).

### 4.3. Hydrolysis and Monosaccharides Compositional Determination of the Polysaccharide

One milligram of polysaccharide was hydrolyzed with 6N HCl at 80 °C in a heating block for 6–8 h. The mixture was cooled and evaporated to remove the acid, resuspended in milli-Q water and passed through a Millipore-GX nylon membrane before analysis. Monosaccharides of polysaccharide hydrolysates were separated on a high-performance anion-exchange chromatographic (HPAEC) system (Dionex, Sunnyvale, CA, USA) and an anion-exchange column (Carbopac PA-10, 4.6250 mm). The analysis of monosaccharides was carried out at an isocratic NaOH concentration of 18 mM at ambient temperature.

### 4.4. Animal Management and Administration

APP/PS1double transgenic mice (No. 005864, Jackson Laboratory, Bar Harbor, ME, USA) express a chimeric mouse/human APP (Mo/HuAPP695swe) and a mutant human presenilin 1 (PS1-dE9) both directed to CNS neurons [35]. They were bred in standard animal house (female C57BL/6 mice and male APP/PS1 mice). Animals were housed under 24 ± 1 °C temperature and 55–65% humidity with a 07:00–19:00 light-dark cycle. The Institutional Animal Care and Use Committee at the National Research Institution of Chinese Medicine approved the animal protocol (IACUCNo: 103-417-2 (103.12.26), 104-417-1 (103.12.21) and 105-417-1 (104.08.10)). Guide for the Care and Use of Laboratory Animals (NIH) was followed for all experimental procedures involving animal and their care.

### 4.5. Metabolic Stress Induction

HFSTZ Mice were created by feeding a high-fat diet and injecting single-dose STZ [3,4,5]. Briefly, 10 week-old mice were fed a HFD (60% energy from fat; TestDiet, St. Louis, MO, USA) with water *ad libitum*. For control, a normal chow diet (NCD; MF-18, Oriental Yeast Co. Ltd., Tokyo, Japan) was used. Mice on HFD also received intraperitoneal injections of STZ (50 mg/kg, in 0.1 M citrate buffer, pH 4.5) 2 weeks after HFD initiated (HFSTZ group). On the contrary, NCD mice were injected with vehicle (0.1 M citrate buffer, pH 4.5). The HFD continued for 11 weeks of HFD manipulation. To evaluate the effect of APS on ameliorating the impairment in HFSTZ mice, APS (500 mg/kg) was orally administrated twice per day for 7 weeks after 4 weeks of HFD administration. The body weight and blood glucose were recorded every week after dietary manipulations except the week for STZ injection.

### 4.6. Blood Glucose Analysis and Oral Glucose Tolerance Test

After fasted for 16 h, Oral Glucose Tolerance Tests (OGTTs) were performed. The mice were then given a glucose solution (3 g/kg; Sigma Aldrich, St. Louis, MO, USA) by oral gavage. Blood is drawn at intervals of 30 min for measurement of glucose. Blood glucose was measured using a glucometer (Bioptik Technology, Taipei, Taiwan).

### 4.7. Tissue Sample Collection

On the end of dietary manipulations, mice were deep anesthetized by injected with 80 mg/kg sodium pentobarbital intraperitoneally. Blood sample was collected by the cardiac puncture and then sacrificed by transcardial perfusion with 50 mL saline. Brain, liver and epididymal fat tissues were collected. 

### 4.8. Leptin and Insulin Analysis

Serum leptin were measured by ELISA (BioVision, Miltipas, CA, USA). Plates were read at a wavelength of 450 nm. Serum insulin levels were determined with an Insulin HTRF High Range kit (Cisbio, Codolet, France). The fluorescence intensities were measured on a SpectraMax M5 microplate reader (Molecular Devices, Sunnyvale, CA, USA). Homeostasis model assessment of insulin resistance scores were calculated as: HOMA-IR = fasting blood glucose (mM) × fasting insulin (U/mL/22.5). 

### 4.9. Hepatic Triglyceride Analysis

Triglyceride (TG) in liver homogenate (100 mg/mL, 5% NP-40) was measured with a TG quantification kit (BioVision, Milpitas, CA, USA). Plates were read at a wavelength of 570 nm using the TECAN GENios plate reader.

### 4.10. Measurement of Aβ

The cerebral cortex was homogenized in ice-cold homogenization buffer (10 mM Tris HCl, 1 mM ethylenediaminetetraacetic acid (EDTA), 0.25 M sucrose, and 1 mM phenylmethylsulfonyl fluoride (PMSF)) containing protease inhibitor cocktail tablets (Roche Diagnostics, Indianapolis, IN, USA). An equal volume of sodium dodecyl sulfate (SDS) solution (1%, PBS with) was added to the homogenate. After centrifugation, the supernatant was collected (i.e., soluble Aβ). The insoluble pellet was re-suspended in 3 M guanidine HCl for 4 h and centrifuged, and the supernatant was collected (i.e., insoluble Aβ). The level of Aβ was determined using Aβ40 and Aβ42enzyme-linked immunosorbent assay (ELISA) kits (Life Technologies, Carlsbad, CA, USA). The absorbance was measured at 450 nm using a TECAN plate reader (Sunrise, UK).

### 4.11. Histological Analysis

Liver and epididymal fat tissues were post fixed in 4% formaldehyde at 4 °C overnight. Paraffin embedded epididymalfat tissues sections (5 µm) were stained with HE. Representative images of HE-stained sections were shown. The size of adipocyte was measured by Image J *S*oftware (National Institutes of Health, Bethesda, MD, USA).

### 4.12. Senile Plaques Staining and Immunohistochemistry

Senile plaques were stained with 0.01% BSB as previously described [3,36]. To analyze the activation of plaque associated astrocyte and microglia, BSB-stained brain sections were further incubated with primary antibodies (i.e., mouse anti-GFAP antibody (1:500, Millipore, Temecula, CA, USA) or goat anti-Iba-1 antibody (1:300, abcam)) at 4 °C overnight; washed with PBS; incubated with fluorescein isothiocyanate-donkey anti-mouse IgG and Alexa Fluor 647-donkey anti-goat IgG (1:300, Jackson ImmunoResearch, West Grove, PA, USA) for 2 h. Slice was mounted with Aqua Poly/Mount (Polysciences, Inc., Warrington, PA, USA). Fluorescent images of BSB staining were taken using a Zeiss LSM 780 confocal microscope (Carl Zeiss Microscopy, Jena, Germany).

### 4.13. Plaque Quantification

Plaque quantification was performed in 11 z-stacked images compiled with maximum intensity projection spanning 10 µm. The number and size of plaques were determined from the images of 20× and 40× magnification, respectively. The size of senile plaques was measured by ImageJ software. Threshold of Images were set by fluorescence at 20–255 and size at >30 µm^2^. The images were converted to binary images for quantification. Plaque diameter was measured as the greatest linear distance between any two points on the perimeter (Feret’s diameter). Two serial sections including the middle region of the hippocampus (−1.58 to −1.94 mm relative to bregma) were analyzed for each mouse.

### 4.14. Quantification of GFAP Intensity Surronding Plaque and Plaque-Associated Iba-1 Intensity

Fluorescent images co-staining with GFAP and BSB at 40× magnification were used for GFAP quantification. The relative fluorescence intensity of GFAP in the vicinity of plaques was quantified using Metamorph software. None plaque associated area with threshold level 30–255, without shade correction was used as reference region. Representative BSB-images were set as 10-µm depth with maximal projection. Binary images were converted with threshold level 20–255. A circle surrounding plaques with diameter of 8 times the Feret’s diameter of plaque was delimited and the immunoreactivity of GFAP in this circle area was determined. 

Fluorescent images of Iba-1 immunostaining with BSB-stained senile plaques were taken at 40× magnification. The relative fluorescence intensity of plaque associated Iba-1 was quantified using Metamorph software. None plaque associated area with threshold level 30–255, without shade correction was used as reference region. 

The overlapping plaques were excluded from analysis. Two serial sections including the middle region of the hippocampus (−1.58 to −1.94 mm relative to the bregma) were analyzed for each mouse.

### 4.15. Nesting Behavior

Mice were individually housed for 5 h, then a nestlet pressed-cotton square (Ancare, Canterbury, Kent, UK) was put into each cage 1 h before the dark cycle started. Nest construction was scored using a 5-point scaling system 16 h later [34]. Nest score of 1 to 5 indicates a >90%, >60%, >30%, >10% and <10% intact nestlet, respectively, and the nest reaching score 5 has an obvious crater. The duration required for constructing a nest reaching scored 5 was recorded with a recording interval for 2 h.

### 4.16. Statistical Analysis

GraphPad Prism (GraphPad, San Diego, CA, USA) was used to perform statistical analysis. All values are mean ± SEM. Statistical analyses were performed using unpaired Student’s *t*-tests for two groups and one-way analysis of variance (ANOVA) followed by Tukey’s honest significant difference (HSD) post hoc test for the groups bigger or equal to 3. Analysis of nesting behavior scores was performed using the non-parametric Kruskal-Wallis test followed by the Mann-Whitney *U* test.

## 5. Conclusions

The results of this study support that APS may be used to ameliorate peripheral metabolic stress, vascular inflammation and neuroinflammation thereby promote AD-related pathology, although Aβ-related pathologies were not altered.

## Abbreviations

Aβ	amyloid-β
AD	Alzheimer’s disease
ADL	activity of daily living
ANOVA	analysis of variant
APP	amyloid precursor protein
AICD	APP intracellular domain
APP/PS1	APPswe/PS1ΔE9
AUC	area under the curve
APS	Astragalus-polysaccharides
BBB	blood-brain barrier
BSB	1-bromo-2,5-bis-(3-hydroxycarbonyl-4-hydroxy) styrylbenzene
CNS	central nervous system
EDTA	ethylenediaminetetraacetic acid
ELISA	enzyme-linked immunosorbent assay
GFAP	glial fibrillary acidic protein
HE	hematoxylin and eosin
HFD	high-fat diet
HFSTZ	HFD and low dose injection of STZ
HOMA-IR	homeostasis model assessment of insulin resistance
Iba-1	ionized calcium-binding adaptor molecule-1
NCD	normal chow diet
OGTT	oral glucose tolerance test
PMSF	phenylmethylsulfonyl fluoride
STZ	strptozotocin
SDS	sodium dodecyl sulfate
TCM	traditional Chinese medicine
TG	triglyceride
